# Dataset on social capital and knowledge integration in project management

**DOI:** 10.1016/j.dib.2020.105233

**Published:** 2020-02-04

**Authors:** Mehmet Ali Ekemen, Harun Şeşen

**Affiliations:** European University of Lefke, Turkey

**Keywords:** Social capital, Knowledge integration, Organizational behaviour, Project management

## Abstract

This research aimed at gaining insight into the behavioural dimensions of projects by recognizing the social capital and the ability of project leaders to incorporate knowledge. The concept and three dimensions of social capital theory, namely structural, relational and cognitive, serve as the basis of the design in this research. The theoretical framework based on the interaction between social capital and the integration of knowledge. A survey was conducted to assess the latent variables of social capital of a project leader on the latent variable of knowledge integration capability. The research carried out with a sample of project management professionals (n = 316), provided project members’ perceptions on the behavioural attributes of project leaders. Structural equation modelling demonstrated the significant positive effect of structural and relational social capital on the integration of knowledge in the project management, but no significant relationship established between cognitive social capital and knowledge integration.

**Table:** Specifications Table

Subject	Strategy and Management
Specific subject area	Social Capital, Knowledge Integration
Type of data	Tables and Figure
How data were acquired	Web-based survey
Data format	Raw and Analyzed
Parameters for data collection	Data were collected from project management professionals in Turkey
Description of data collection	Data were collected using web-based self-administrated questionnaire from 316 valid samples
Data source location	Turkey
Data accessibility	Data are included in this article

**Table:** 

**Value of the Data** • The analysis focused on the multidimensional nature of social capital which is confined in the field of empirical research. The dataset can be used to extend the research to show how this multidimensional nature of social capital and knowledge integration impacts on the project team performance. • The dataset can be used as a reference to undertake comparative studies in project management among the contexts of other countries. • The dataset can be used to further analyse the role of demographics and project characteristics in the relationship between social capital and the incorporation of knowledge in project teams. • The dataset offers measures for social capital and knowledge integration which are both latent (unobserved) variables and difficult to measure. The measurement model of the analysis helps to develop similar frameworks for extending research in this field.

## 1 Data

The survey sample is composed of project management professionals in Turkey. SEM is ideal for the multidimensional, abstract analysis and validation of the overall model fit of the social capital theory, yet requires a large number of samples. Depending on the sample size literature, the general rule for minimum sample size is 100, with the option of 200 or 5 to 20 times the estimated number of variables, whichever is greater [1,2]. The optimal sample size goal of this analysis was to reach 200, which included the minimum required sample size and the minimum recommended factor ratio.

A convenience sample of participants voluntarily accessed a survey hyperlink via invitation email sent to 500 Project Management Professionals. The participants were not restricted by particular types of organizations, sectors or organizational sizes. Given the wide range of the sampling process, it was important to monitor respondents with prior project management expertise in order to limit external threats to survey responses. The external threat of validity based on the ideal characteristics of the sample has been reduced by participants with involvement of a project team in the last 3 years, in order to provide accurate data. Participants decided online to take or not before getting access to the survey. Participants could leave the survey at any point. The participation to the survey was anonymous because it was accessible through a third-party online survey software system, with the hyperlink sent through email. The questionnaire was consisting of 19 items in 5 point Likert scale (0 = strongly disagree to 5 = strongly agree) to measure variables and 6 items to capture Demographics and Project Characteristics, given in supplementary files. A total of 316 survey responses resulted in a response rate of 63%. Table 1 provides aggregate data of the demographics and project characteristics obtained from the sample (n = 316).

**Table 1:** Demographics and project characteristics.

Item	N	%
**Gender**	**316**	**100%**
(1) Male	212	67%
(2) Female	104	33%
**Education**	**316**	**100%**
(1) High School	–	–
(2) Associate Degree	4	1%
(3) Bachelor Degree	145	46%
(4) Master Degree	161	51%
(5) PhD	6	2%
**Age**	**316**	**100%**
(1) Less than 30	3	1%
(2) 30–39	42	13%
(3) 40–49	138	44%
(4) 50–59	122	39%
(5) More than 59	11	3%
**Have been Project Manager?**	**316**	**100%**
(1) Yes	83	26%
(2) No	233	74%
**Experience**	**316**	**100%**
(1) Less than 5 years	12	4%
(2) 5–10	21	7%
(3) 11–15	105	33%
(4) 16–20	81	26%
(5) More than 20 years	97	30%
**Total No of Project Participated**	**316**	**100%**
(1) Less than 5	18	6%
(2) 5–10	13	4%
(3) 11–15	73	23%
(4) 16–20	99	31%
(5) More than 20	113	36%

The survey instrument included 19 items designed to measure the characteristics of three independent latent variables and one dependent latent variable. The data was tested for common method bias by employing Harman one factor analysis [3] and obtained 30% of the variance explained by single factor which is less than %50 of benchmark. The validity and reliability measures of instrument items are shown in Table 2.

**Table 2:** Validity and reliability measures.

Model Construct	Mean	SD	Cr. Alpha	Average Variance Extracted (AVE)	Composite Reliability (CR)	Factor Loading (EFA)	Factor Loading (CFA)
Structural Social Capital [4]			.82	.55	.86		
SSC1	4.14	.78				.80	.69
SSC2	4.08	.81				.79	.74
SSC3	3.67	.92				.70	.73
SSC4	4.37	.72				.63	.61
SSC5	4.11	.82				.77	.74
Relational Social Capital [5]			.87	.64	.88		
RSC1	4.26	.78				.82	.89
RSC2	4.16	.81				.80	.91
RSC3	4.15	.86				.78	.58
RSC4	4.13	.86				.80	.65
Cognitive Social Capital [6]			.82	.73	.89		
CSC1	2.73	1.33				.79	.63
CSC2	3.25	1.28				.87	.85
CSC3	2.93	1.30				.89	.86
Knowledge Integration [7,8]			.86	.51	.88		
KI1	4.24	.81				.57	.50
KI2	4.11	.87				.70	.51
KI3	4.09	.83				.58	.66
KI4	4.19	.78				.82	.76
KI5	4.27	.71				.71	.73
KI6	4.02	.81				.81	.81
KI7	4.33	.75				.77	.78

## 2 Experimental design, materials, and methods

### 2.1 Design

The aim of this research was to examine the social processes of knowledge integration by examine the social capital of project management. The social factors included structural, relational and cognitive aspects of the social capital of a project leader, and how these social aspects contribute to the incorporation of knowledge.

Social capital theory is a theoretical framework used to describe the potential links between multiple social capital dimensions and knowledge integration [9]. Nahapiet and Ghoshal [6] characterized social capital as the total of present and future resources integrated in, accessible and generated from the chain of connections possessed by a person or a social unit and offered three different dimensions as structural, relational and cognitive. Relationships and information access describe the structural elements of social capital. The relational elements described the advantages of connections and how they control behaviour. The cognitive elements of social capital are based on mutual communication experiences.

It is the duty of the project leader to combine different knowledge of various disciplines [10] in order to achieve the required project performance. Knowledge integration involves the implementation of knowledge [7], the consolidation of diverse knowledge [11], or a collaborative mechanism which transfers individual knowledge and embraces individual knowledge into new knowledge [12]. A project team's capacity to combine knowledge and accomplish project goals is a function of the team resources provided by the social capital of the project leader. The research design assumes that the social capital of a project leader positively contributes to knowledge integration by understanding different members within the social structures and acknowledging the team advantage of all members within the social structure [13].

### 2.2 Method

The measures given in the provided dataset were gathered from primary research and an online survey questionnaire. The questionnaire includes items related to three dimensions of social capital (structural, relational, cognitive) and knowledge integration in project teams. It also includes questions related to demographics (gender, age, education) and project characteristics (have been a project manager, experience, total number of projects participated). The survey was administered to sample of 316 project management professionals in Turkey with provided written informed consent.

**Fig. 1:**
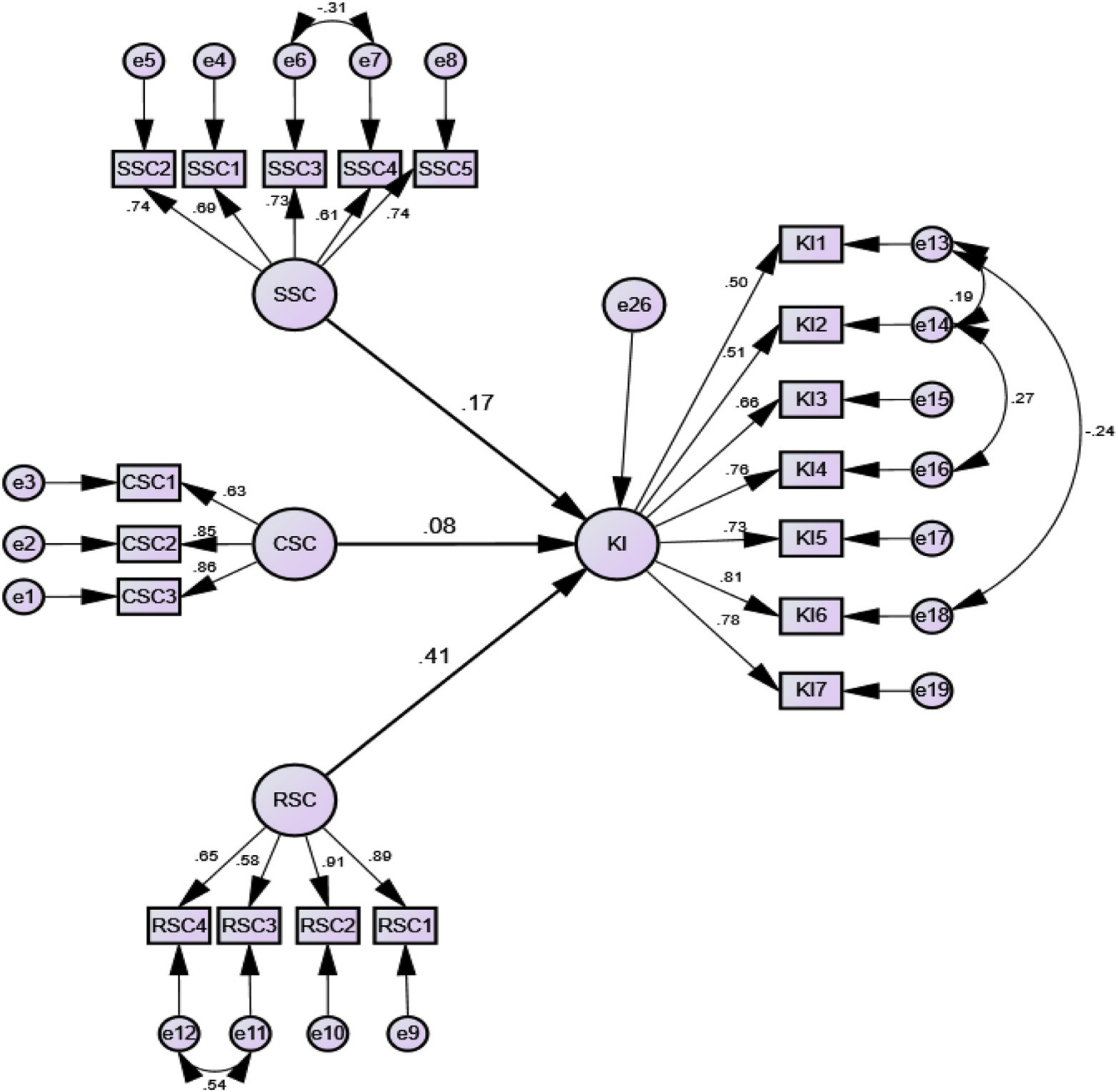
Measurement and structural model.

Social capital is multidimensional and endogenous which makes it more difficult to observe directly. Therefore, the survey approach and the use of SEM as a tool for data analysis were appropriate for evaluating the latent variables in the model. The use of Structural Equation Modelling permits both the measurement and evaluation of a priori model components. The analysis of measurement model provided a way to link multiple observable indicators to each latent in order to understand unobservable variables, therefore the dimensions of the social capital investigated [14]. The structural part of the model provided a framework for an overall analysis of social capital and its relationship with knowledge integration. Since covarying interrelationship of the structural, relational, and cognitive social capital variables exist, SEM would be the best option to integrate all these relationships at once. Fig. 1 highlights both the measurement model and the structural model.

The analysis identified positive significant impact of structural and relational social capital on knowledge integration, but no significant relationship found between cognitive social capital and knowledge integration. Data analysis outcomes are shown in Table 3.

**Table 3:** Regression analysis outcomes.

			Standardized Beta	S.E.	C.R.	p
KI	<---	SSC	.17	.05	2.68	.01
KI	<---	CSC	.08	.02	1.34	.17
KI	<---	RSC	.41	.04	5.59	.00

## Conflict of Interest

The authors declare that they have no known competing financial interests or personal relationships that could have appeared to influence the work reported in this paper.
