# Supplementary material for: Dataset on social capital and knowledge integration in project management
Source: Data Brief. 2020 Feb 4;29:105233. doi: 10.1016/j.dib.2020.105233 (PMC7016259; doi:10.1016/j.dib.2020.105233)
Supplement: Multimedia component 2 [file mmc2.docx]

Consent to Participate Letter

Hello,

My name is Mehmet Ali Ekemen and I invite you to participate in a research study aims to understand the role of social capital in project teams. This study is being conducted as a part of academic research.

This research study is about the ability of project leaders to integrate knowledge within a project team and the results of this study will help to better understand how a project leader’s social capital is related to knowledge integration within a team. Your participation will involve completing a 25 questions survey that should take approximately 15 minutes to complete and will be highly appreciated.

Your participation in this study is completely voluntary. There are no foreseeable risks associated with this project. However, if you feel uncomfortable answering any questions, you can decline to participate, you can skip any questions, or you can withdraw from the survey at any point during the survey.

Your survey responses will be strictly confidential and data from this research will be reported only in aggregate. If you have questions at any time about the survey or the procedures, or you are interested in a copy of the final results, you may contact me by email at mekemen@eul.edu.tr. You may print or keep a copy of this consent form for your records.

Thank you very much for your time and support. While no compensation is provided for your voluntary participation, please know that I greatly appreciate your time and effort to complete this quick 15 minutes’ survey.

If you meet the survey participation requirement of working on a project team of a completed project within the past 3 years, please start with the survey now by clicking on the link provided.

Sincerely,

Mehmet Ali Ekemen

Please pick a project team you participated in as a team member and answer **ALL** of the following survey questions relative to your experiences on this project team you have chosen.

**Survey Questions**

| No | Structural Social Capital | Strongly Disagree | Disagree | Undecided | Agree | Strongly Agree |
| --- | --- | --- | --- | --- | --- | --- |
| 1 | I had access to the project manager when I needed him/her |  |  |  |  |  |
| 2 | The project leader was able to get external information quickly |  |  |  |  |  |
| 3 | The project leader encouraged communication with other team members throughout the project |  |  |  |  |  |
| 4 | The project manager recognized and sought out the knowledge, skills and abilities I brought to the project team |  |  |  |  |  |
| 5 | The project leader was able to acquire resources for the project and the team members |  |  |  |  |  |
|  | Rational Social Capital | Strongly Disagree | Disagree | Undecided | Agree | Strongly Agree |
| 6 | The project leader encouraged the project team to “think outside the box” and take risks |  |  |  |  |  |
| 7 | I had a high degree of trust in the project leader because he/she acted in the best interest of the project and the project team |  |  |  |  |  |
| 8 | I had a high degree of trust that the project leader had the competence to perform his/her role as the project leader |  |  |  |  |  |
| 9 | I had a high degree of trust that the project leader had the capability to perform his/her role as the project leader |  |  |  |  |  |
|  | Cognitive Social Capital | Strongly Disagree | Disagree | Undecided | Agree | Strongly Agree |
| 10 | The project leader shared important project goals, tasks, and documents with the project team |  |  |  |  |  |
| 11 | The project leader’s goals and objectives were the same as the project team’s goals and objectives |  |  |  |  |  |
| 12 | The project leader held routine meetings with the project team |  |  |  |  |  |
|  | Knowledge Integration | Strongly Disagree | Disagree | Undecided | Agree | Strongly Agree |
| 13 | I had access to project information and project data when I needed it |  |  |  |  |  |
| 14 | A common system or database was used by team members to store information in a common location that was available to the project team |  |  |  |  |  |
| 15 | The project leader communicated knowledge and information related to the project challenges, needs, and/or changes on a regular basis |  |  |  |  |  |
| 16 | I could not have completed my project tasks/ responsibilities without knowledge and information from other members of my team |  |  |  |  |  |
| 17 | The project leader allowed for decision making at the appropriate level |  |  |  |  |  |
| 18 | The project team developed new ideas that were incorporated into the project tasks and decisions |  |  |  |  |  |
| 19 | The project leader brought together the project team to share new information or specialized knowledge that was relevant to the project |  |  |  |  |  |

You are almost finished. This final section is associated with personal and professional information. Please note that all information is private and confidential and will only be used in aggregate.

Q20. Please indicate your gender:

1. Male
2. Female

Q21. Please indicate your age range:

1. Less than 30
2. 30 – 39
3. 40 – 49
4. 50 – 59
5. More than 59

Q22. Please indicate the highest degree of education you obtained:

1. High School
2. Associate Degree
3. Bachelor Degree
4. Master Degree
5. PhD

Q23. Please indicate if you have been a project manager:

1. Yes
2. No

Q24. Please indicate how many years of experience you have in project teams:

1. Less than 5 years
2. 5 – 10
3. 11 – 15
4. 16 – 20
5. More than 20 years

Q25. Please indicate the total number of projects you participated:

1. Less than 5
2. 5 – 10
3. 11 – 15
4. 16 – 20
5. More than 20
